# Different patterns of epileptiform-like activity are generated in the sclerotic hippocampus from patients with drug-resistant temporal lobe epilepsy

**DOI:** 10.1038/s41598-018-25378-9

**Published:** 2018-05-08

**Authors:** Selvin Z. Reyes-Garcia, Carla A. Scorza, Noemi S. Araújo, Nancy N. Ortiz-Villatoro, Anaclara Prada Jardim, Ricardo Centeno, Elza Márcia Targas Yacubian, Jean Faber, Esper A. Cavalheiro

**Affiliations:** 1 Departamento de Neurologia e Neurocirurgia, Escola Paulista de Medicina, Universidade Federal de São Paulo, São Paulo, Brazil; 2 Departamento de Ciencias Morfológicas, Facultad de Ciencias Médicas, Universidad Nacional Autónoma de Honduras, Tegucigalpa, Honduras

## Abstract

Human hippocampal slice preparations from patients with temporal lobe epilepsy (TLE) associated with hippocampal sclerosis (HS) are excellent material for the characterization of epileptiform-like activity. However, it is still unknown if hippocampal regions as *cornu Ammonis* (CA) 1, CA3 and CA4, generate population epileptiform-like activity. Here, we investigated epileptiform activities of the subiculum, CA1, CA2, CA3, CA4 (induced by elevation of extracellular potassium concentration) and the dentate gyrus (induced with hilar stimulation and elevation of potassium concentration) from sclerotic hippocampi of patients with drug-resistant TLE. Five types of epileptiform-like activity were observed: interictal-like events; periodic ictal spiking; seizure-like events; spreading depression-like events; tonic seizure-like events and no activity. Different susceptibilities to generate epileptiform activity among hippocampal regions were observed; the dentate gyrus was the most susceptible region followed by the subiculum, CA4, CA1, CA2 and CA3. The incidence of epileptiform activity pattern was associated with specific regions of the hippocampal formation. Moreover, it was observed that each region of the hippocampal formation exhibits frequency-specific ranges in each subfield of the sclerotic human tissue. In conclusion, this study demonstrates that epileptiform-like activity may be induced in different regions of the hippocampal formation, including regions that are severely affected by neuronal loss.

## Introduction

Temporal lobe epilepsy is one of the most common types of focal epilepsies in adults. In over 30% of the patients with temporal lobe epilepsy (TLE), seizures cannot be controlled with currently available antiepileptic drugs (AED) and approximately 17% of these patients will die as a result of their epilepsy^1^. Hippocampal sclerosis (HS) is the most frequent histopathological finding in refractory TLE^2,3^. Temporal lobe epilepsy associated with HS is often progressive with worsening of seizures, impairment of cognitive function, psychiatric disorders and is accompanied by significant morbidity and mortality^4–6^. In this scenario, resective surgery has been recognized as an effective treatment for pharmacoresistant TLE/HS^7–10^.

The pattern of cell loss in HS is highly heterogeneous and enormous efforts have been made to classify specific patterns of neuronal loss and correlate the subtypes with postsurgical outcome. Moreover, some patients with TLE benefit from excision of the ictogenic focus, suggesting that epileptic tissue is responsible for both hyperexcitability and/or drug resistance^11^. However, one third of the surgically treated patients presented unfavorable results^12^. Therefore, TLE/HS is a heterogeneous condition and there are gaps in our understanding of its pathophysiological mechanisms, natural history and progression.

*In vitro* studies of human tissue have been conducted to explore the population epileptiform-like activity and understand how ictal events are generated in the sclerotic hippocampus. In this context, human hippocampal slice preparations from TLE/HS patients have been used in the evaluation of epileptiform-like activity in the subiculum, *Cornu Ammonis* (CA) 2 and dentate gyrus, areas that appear to be more resistant to neuronal loss in hippocampal sclerosis^3,13–16^. Spontaneous interictal-like activity was observed in the subiculum and the CA2^17–22^. In the dentate gyrus, different patterns of epileptiform activity may be induced by electrical stimulation and elevation of extracellular levels of potassium^11,23^. The underlying mechanisms involved in the generation of epileptiform activity seem to be different for each hippocampal area^18–20,23,24^, although defects in GABAergic transmission and enhanced glutamatergic signaling have been suggested as playing causative roles^25^. However, epileptiform-like population activities in other subfields (CA1, CA3, CA4) of the human sclerotic hippocampus are still unknown.

Here, using *in vitro* electrophysiology, we assessed slices of the human hippocampus, resected from patients with drug resistant TLE and evaluated the incidence of ictal activity in different regions of the hippocampal formation. We demonstrate that distinct patterns of epileptiform-like activity are generated by different hippocampal subfields. The incidence of epileptiform population activity pattern was associated with specific regions of the hippocampal formation: interictal-like events were predominantly induced in the CA3 and CA4 regions, periodic ictal spiking in the subiculum as well as in the CA2, and seizure-like events in the dentate gyrus. Moreover, we observed that each region of the hippocampal formation processes the electrical activity in a specific way, since a frequency-specific range of epileptiform activity was observed for each hippocampal area.

## Results

### The type of epileptiform-like activity is associated with different subfields of the hippocampal formation

**Figure 1:**
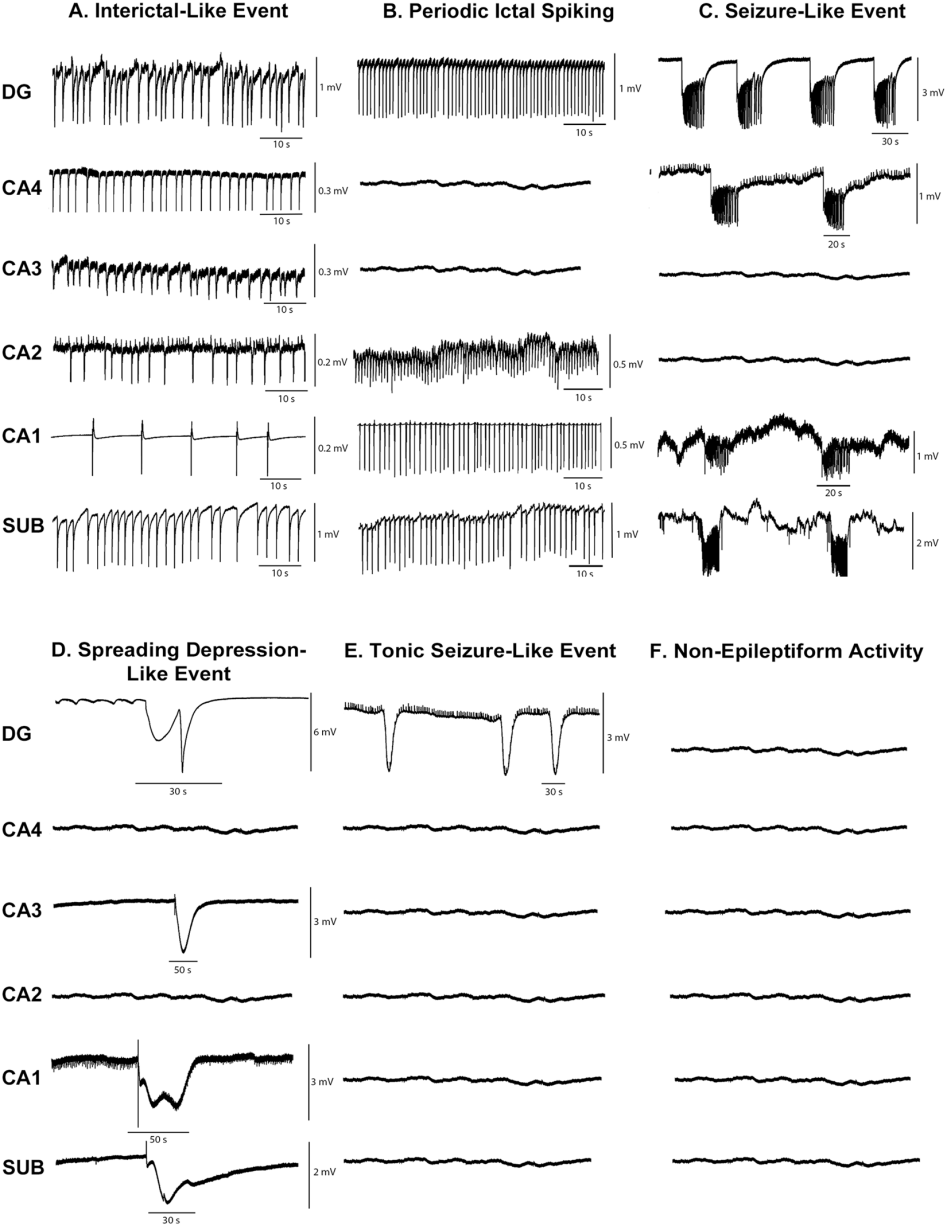
Epileptiform-like activity in hippocampal formation. Representative recordings of the different patterns of epileptiform-like activity in different regions of hippocampal formation. (**A**) Interictal-like events. (**B**) Periodic ictal spiking. (**C**) Seizure-like event. (**D**) Spreading depression-like events. (**E**) Tonic seizure-like events. (**F**) Non-epileptiform activity. Isoelectric recordings indicate that non-epileptiform activity was observed. Calibration bars for amplitude and time are given in each recording on the right.

**Figure 2:**
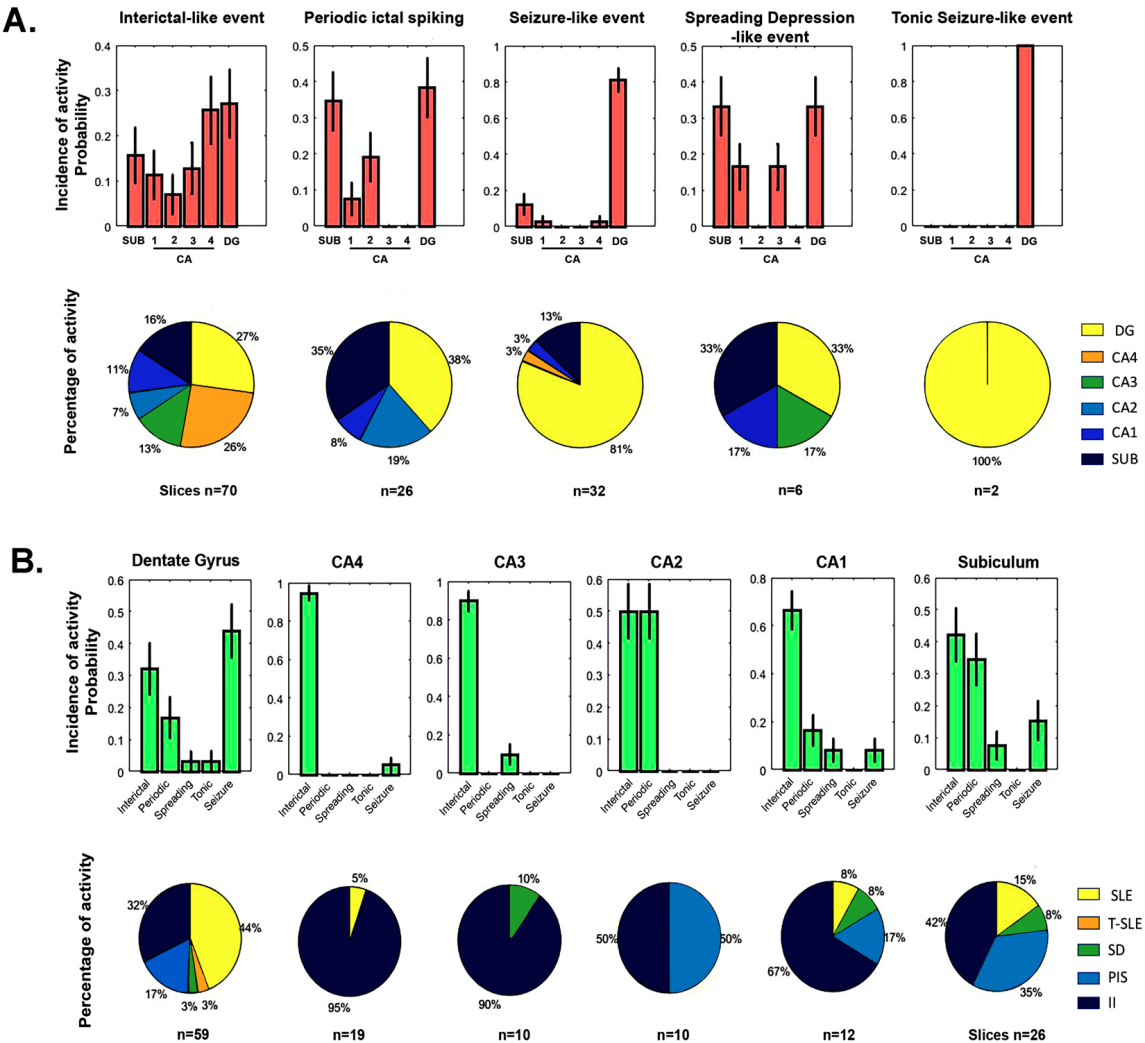
Distribution of epileptiform activity. (**A**) Conditional probability distribution of generated specific epileptiform-like activity within the hippocampal subfields *P* (epileptiform activity|hippocampal area). (**B**) Conditional probability distribution of specific hippocampal formation area to generate epileptiform activity *P* (hippocampal area|epileptiform activity). Bar graphs represent the mean probability with the corresponding standard deviation. Circular graphs represent the absolute frequencies. SLE: Seizure-like events; T-SLE: Tonic seizure-like events; SD: Spreading depression-like events; PIS: Periodic ictal spiking; II: interictal-like events.

Electrophysiological recordings were collected from 143 slices from 30 human hippocampal specimens. Epileptiform activity was induced in the dentate gyrus by hilar electrical stimulation and continuous perfusion with artificial cerebrospinal fluid (aCSF) containing 10–12 mM [K^+^] (high K-aCSF). In the subiculum and hippocampal subfields, electrical stimulation was not necessary since only high levels of K-aCSF were sufficient to provoke epileptiform activity (see details in Methods). Figure 1 shows the representative traces of epileptiform-like activity in different regions of the hippocampal formation. Five types of epileptiform activity were observed: (a) interictal-like events (number of slices, n = 70; 49.0%); (b) periodic ictal spiking (n = 26; 18.2%); (c) seizure-like events (n = 32; 22.4%); (d) spreading depression-like events (n = 6; 4.2%); (e) tonic seizure-like events (n = 2; 1.4%) and; (f) non-epileptiform activity (n = 7; 4.9%). Different susceptibility to generate epileptiform activity among hippocampal regions was observed, whereby the dentate gyrus was the most susceptible region (n = 59; 43.4%), followed by the subiculum (n = 26; 19.1%), CA4 (n = 19; 14%), CA1 (n = 12; 8.8%), CA2 (n = 10; 7.4%) and CA3 (n = 10; 7.4%). In addition, it was possible to establish an association between the type of epileptiform activity and the hippocampal subfield (χ^2^ = 54.68, p < 0.0001). Adjusted residual values indicated the following associations: interictal-like activity with the CA3 and CA4 regions; periodic ictal spiking with the subiculum as well as CA2; and seizure like-events with the dentate gyrus. The incidences of epileptiform-like activity were observed to be as follows: interictal-like events, in CA4 (n = 18; 25.7%) and in CA3 (n = 9; 12.9%); periodic ictal spiking in the subiculum (n = 9; 34.6%) and in CA2 (n = 5; 19.2%); seizure like-events in the dentate gyrus (n = 26; 81.3%) (Fig. 2).

### Electrophysiological characterization of the different patterns of epileptiform activity

Interictal-like events, which resemble interictal spiking recorded with intracerebral electroencephalographic (EEG) in patients^19,24^, were observed in all subfields of the hippocampal formation: dentate gyrus (number of slices, n = 19; 27.1%), CA4 (n = 18; 25.7%), subiculum (n = 11; 15.7%), CA3 (n = 9; 12.9%), CA1 (n = 8; 11.4%), CA2 (n = 5; 7.1%). The highest amplitudes were observed in both the dentate gyrus and the subiculum, followed by the CA4, CA3, CA2 and CA1 (Table 1). Significant regional differences in amplitude of the interictal-like events were encountered (H = 44.77, p < 0.0001); *post hoc* Dunn’s test indicated differences between the dentate gyrus and CA1, CA2, CA3 subfields and differences between the subiculum and CA1, CA2, CA3, CA4. Regarding the duration of these events, the highest mean durations were observed in both the dentate gyrus and the subiculum, followed by the CA4, CA3, CA2 and CA1; significant differences were observed among hippocampal regions (H = 17.76; p = 0.0033), *post hoc* Dunn’s test indicated differences between the subiculum and CA1. Regarding the number of events per minute, the CA2, subiculum and the dentate gyrus presented the highest rates of interictal-like events, followed by those in the CA4, CA3 and CA1; significant differences were observed (H = 22.75; p = 0.0004), *post hoc* Dunn’s test demonstrated differences between the subiculum and CA1, CA3 (Table 1).

**Table 1:** Parameter values of the different patterns of epileptiform activity in hippocampal formation subfields.

Hippocampal formation subfield	DG	CA4	CA3	CA2	CA1	Subiculum	P-value
Epileptiform-like activity							
**Interictal-like event**							
Slices (n)	19	18	9	5	8	11	
Events/min^b^	21.7 ± 1.9	18.0 ± 0.8	15.0 ± 1.1	22.0 ± 1.2	14.0 ± 1.6	21.8 ± 0.9	0.0004
Amplitude (mV)^b^	1.0 ± 0.1	0.3 ± 0.02	0.2 ± 0.04	0.2 ± 0.01	0.2 ± 0.01	0.9 ± 0.1	0.0001
Duration (sec)^b^	0.7 ± 0.1	0.4 ± 0.04	0.4 ± 0.04	0.4 ± 0.1	0.3 ± 0.01	0.7 ± 0.04	0.0033
**Periodic ictal spiking**							
Slices (n)	10	—	—	5	2	9	
Events/min^b^	52.5 ± 2.1	—	—	103.6 ± 5.9	42.5 ± 1.4	48.1 ± 2.4	0.0024
Amplitude (mV)^b^	1.1 ± 0.1	—	—	0.3 ± 0.03	0.4 ± 0.02	0.9 ± 0.01	0.0028
Duration (sec)^b^	0.4 ± 0.03	—	—	0.2 ± 0.03	0.2 ± 0.01	0.12 ± 0.03	0.0004
**Seizure-like events**							
Slices (n)	26	1	—	—	1	4	
Events/min	1.5 ± 0.2	0.7 ± 0.2	—	—	1.5 ± 0.4	0.9 ± 0.2	
Amplitude (mV)^a^	2.6 ± 0.1	0.8 ± 0.1	—	—	0.6 ± 0.1	1.2 ± 0.1	0.0007
Duration (sec)	28.9 ± 3.1	17.2 ± 2.0	—	—	15.6 ± 3.2	19.3 ± 0.8	
**Spreading depression-like event**							
Slices (n)	2	—	1	—	1	2	
Events/min	1	—	1	—	1	1	
Amplitude (mV)	5.3 ± 0.4	—	3.2	—	2.3	1.2 ± 0.1	
Duration (sec)	35.5 ± 3.9	—	57.6	—	105.8	51.1 ± 5.2	
**Tonic seizure-like events**							
Slices (n)	2	—	—	—	—	—	
Events/min	1.1 ± 0.2	—	—	—	—	—	
Amplitude (mV)	2.6 ± 0.54	—	—	—	—	—	

^a^P-values are indicated where significant difference for group were observed using Mann-Whitney-Wilcoxon U test. ^b^P-values are indicated where significant difference for group were observed using Kruskal-Wallis H test. All data are expressed as mean ± SEM.

Periodic ictal spiking, which seems to mimic the activity recorded in the human sclerotic hippocampus using depth-electrodes^26,27^, was induced in the dentate gyrus (n = 10; 38.5%), subiculum (n = 9; 34.6%), CA1 (n = 2; 7.7%) and CA2 (n = 5; 19.2%); this type of activity was not observed in the CA3 and CA4 subfields. The highest amplitude was observed in both the dentate gyrus and the subiculum, followed by that in CA1 and CA2. Significant differences were observed among hippocampal regions (H = 14.07, p = 0.0028); *post hoc* Dunn’s test demonstrated differences between CA2 and the dentate gyrus as well as the subiculum. The longest duration of periodic ictal spiking events was observed in the dentate gyrus followed by that in CA2, CA1 and the subiculum. Significant differences were observed among regions (H = 18.07, p = 0.0004), *post hoc* Dunn’s test demonstrated differences between the dentate gyrus and the subiculum. The number of events per minute was predominantly high in the CA2 region followed by that in the dentate gyrus, subiculum and CA1. Statistically significant differences were observed among regions (H = 14.44, p = 0.0024), *post hoc* Dunn’s test demonstrated differences between CA2 and the dentate gyrus as well as CA1 (Table 1).

Seizure-like events were induced predominantly in the dentate gyrus (n = 26; 81.3%); this pattern of activity was also observed in subiculum (n = 4, 12.5%), CA1 (n = 1; 3.1%) and CA4 (n = 1; 3.1%) but was not observed in the CA2 and CA3 regions. The highest mean amplitude was observed in the dentate gyrus followed by that in the subiculum, CA4 and CA1. Significant differences (Mann-Whitney-Wilcoxon U:0.0; p = 0.0007) were observed between the dentate gyrus and subiculum. The longest duration of seizure-like events was observed in the dentate gyrus followed by that in the subiculum, CA4, and CA1. However, significant differences were not observed (U = 22, p = 0.1232). Regarding the number of events per minute, no significant differences were observed (U = 16, p = 0.1284) (Table 1).

Spreading depression-like events were observed in the dentate gyrus (n = 2; 33.3%), subiculum (n = 2; 33.3%), CA3 (n = 1; 16.7%) and CA1 (n = 1; 16.7%); however, it was not possible to detect significant differences related to electrophysiological parameters (amplitude, rate and duration of events) due to the small sample size (Table 1).

Tonic seizure-like events were encountered in the dentate gyrus (n = 2; 100%), but not in the other hippocampal regions assessed. Amplitude mean in the dentate gyrus was 2.6 ± 0.4 mV, the duration of events was 34.2 ± 2.7 sec and the number of events was 1.1 ± 0.2 events per minute (Table 1).

In summary, interictal-like events were encountered in all hippocampal regions, and the dentate gyrus and subiculum generated the highest amplitudes, duration and rate of events. Periodic ictal spiking demonstrated the highest amplitudes in both the dentate gyrus and subiculum, the longest duration was observed in the dentate gyrus and a strikingly high number of events was observed in the CA2 hippocampal subfield. Seizure-like events demonstrated the highest amplitudes and duration in the dentate gyrus. Regarding spreading depression-like events and tonic seizure-like events, it was not possible to detect significant differences in the parameters assessed due to the small sample sizes (Table 1).

### Histopathological analysis

**Figure 3:**
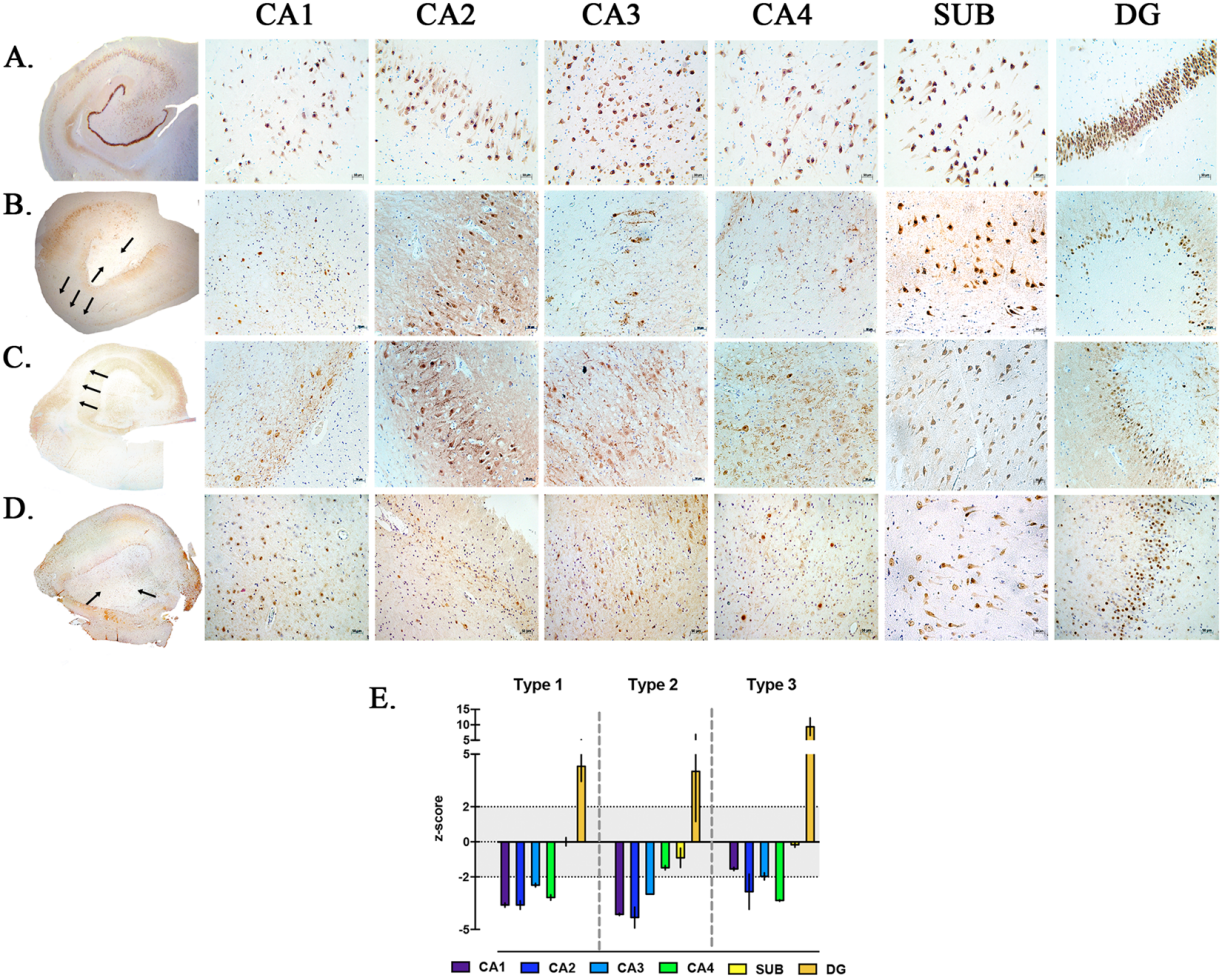
Histopathological patterns of hippocampal sclerosis. (**A**–**D**) NeuN immunostaining of neuronal nuclei. (**A**) No hippocampal sclerosis in control specimen obtained from autopsy without neurological disease. (**B**) ILAE hippocampal sclerosis type 1, note the marked neuronal loss in both the CA1 and CA4 subfields. (**C**) ILAE hippocampal sclerosis type 2, predominant cell loss in the CA1 area. (**D**) ILAE hippocampal sclerosis type 3, most restrict neuronal loss in the CA4 sector. Among all hippocampal sclerosis types, the histological pattern is variable in the dentate gyrus, wherein the granular cell loss and dispersion is predominant. Hippocampal subfields and the dentate gyrus were observed with a magnification of 20×. Arrows indicate regions with severe neuronal loss. (**E**) Measurement of neuronal density in hippocampal subfields, neuronal cell counting was transformed into z-score using values from control hippocampi. Hippocampal sclerosis sectors were determined when cell neuronal counting was <than −2 in z-score values. For the dentate gyrus, granular cell dispersion was considered when width reached >2 in z-score values.

Histopathological analysis of 30 hippocampal specimens at the mid-body level, through NeuN immunostaining, allowed the identification of distinct patterns of neuronal loss which were categorized according to the ILAE classification of hippocampal sclerosis (HS) (Fig. 3). Three different patterns were identified: HS ILAE type 1 was detected in 26 specimens (86%); HS ILAE type 2 in 2 (7%) and HS ILAE type 3 in 2 (7%). Hippocampal neuronal cell densities and histopathological patterns of dentate gyrus are described in Table 2. Differences in cell densities were observed in both CA1 and CA4 when compared among all types of ILAE HS. In the CA1 subfield (H = 8.9, p = 0.0007), the difference between HS type 2 and type 3 was observed using *post hoc* Dunn’s test. In CA4 (F = 7.7. p = 0.0037), *post hoc* Bonferroni’s correction demonstrated the difference between HS type 1 and type 2, as well as between HS type 2 and type 3.

**Table 2:** Clinical and histopathological information of patients with pharmacoresistant temporal lobe epilepsy.

Variables and Categories	Hippocampal Sclerosis ILAE type			P-value
	1	2	3	
**Age (Years)**	35.2 ± 2.4	28.5 ± 17.5	29.5 ± 4.5	
**Gender**				
Female	16	1	2	
Male	10	1	0	
**Initial Precipitating Injury**				
Febrile seizure	8	1	0	
TBI	1	0	1	
Unknown	16	1	1	
Neuroinfection	1	0	0	
**Site of resection**				
Right	14	1	2	
Left	12	1	0	
**Age at epilepsy onset (years)**	12.9 ± 1.7	7.3 ± 4.8	21 ± 3	
**Epilepsy duration (years)**	21.8 ± 2.5	21.3 ± 12.8	8.5 ± 1.5	
**FAS per month**	7 ± 1.8	5 ± 3	4	
**FIS per month**	6.6 ± 1.8	5 ± 3	4	
**SG per month**	1.3 ± 0,5	0	0	
**AEDs**	4.9 ± 0.3	4.5 ± 0.5	4.5 ± 1.5	
**Neuronal Density x 10** ^**−4**^ **/µm** ^2^				
CA1^a^	0.5 ± 0.1	0.2 ± 0.05	1.9 ± 0.1	0.0007
CA2	1.1 ± 0.1	0.9 ± 0.1	1.4 ± 0.1	
CA3	0.8 ± 0.1	0.8 ± 0.1	1.1 ± 0.1	
CA4 ^a^	0.5 ± 0.1	1.4 ± 0.1	0.5 ± 0.1	0.0037
Subiculum	141.3 ± 20.8	125.5 ± 13.4	138.5 ± 3.5	
**Dentate gyrus width GCD (µm)**	155.8 ± 27.6	150.5 ± 26.2	250.3 ± 25.5	
**Dentate gyrus pattern**				
Bilamination	2	0	0	
Dispersion	14	1	2	
Preserved	9	1	0	
Loss	1	0	0	

TBI: traumatic brain injury; FAS: focal aware seizure; FIS: focal impaired awareness seizure; GS: generalized seizure; AEDs: antiepileptic drugs; GCD; granular cell dispersion. Data from histology is presented as neuronal densities × 10^−4^/µm^2^. All data are expressed as the mean ± SEM. ^a^P-values are indicated when significant difference for group were observed using Kruskal-Wallis H test.

**Figure 4:**
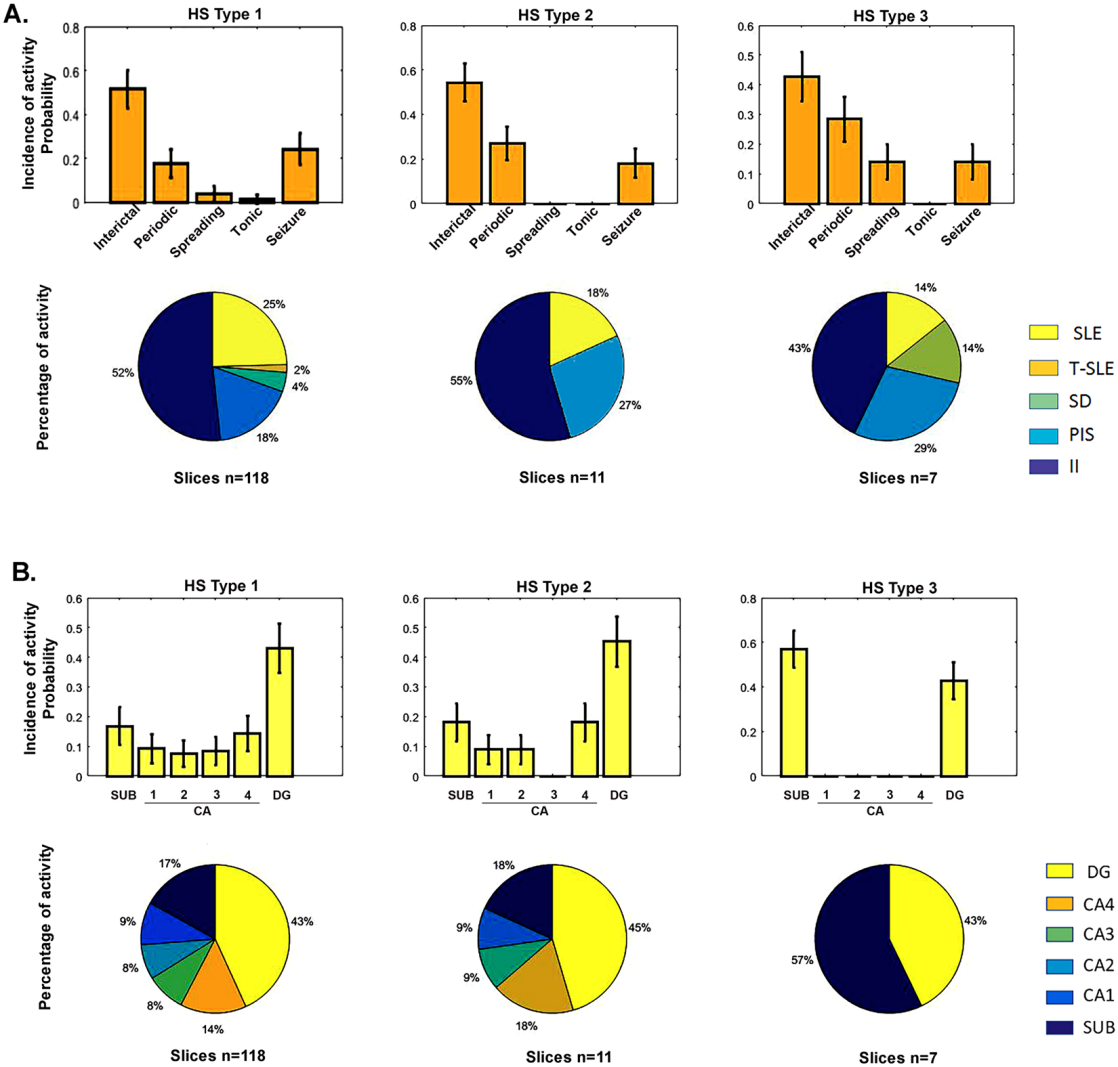
Distribution of epileptiform activity by ILAE HS type. (**A**) Conditional probability distribution of the different HS types to generate specific patterns of epileptiform activity *P* (HS type|epileptiform activity). (**B**) Conditional probability distribution of the different HS types to present epileptiform activity susceptibility in the different hippocampal subfields *P* (HS Type|hippocampal area). Bars graphs represent the mean probability with the corresponding standard deviation. Circular graphs represent the absolute frequencies. SLE: Seizure-like events; T-SLE: Tonic seizure-like events; SD: Spreading depression-like events; PIS: Periodic ictal spiking; II: interictal-like events.

Next, the electrophysiological recordings were categorized according to the type of hippocampal sclerosis. Electrophysiological recordings of 118 slices from HS type 1 were assessed, 11 from HS type 2 and 7 from HS type 3. Regarding HS type 1, five patterns of epileptiform activity were observed: (1) interictal-like events (n = 61; 51.7%); (2) seizure-like events (n = 29; 24.6%); (3) periodic ictal spiking (n = 21; 17.8%); (4) spreading depression-like events (n = 5; 4.2%); and (5) tonic seizure-like events (n = 2; 1.7%). Induction-susceptibility of epileptiform activity was predominant in the dentate gyrus (n = 51; 43.2%), followed by that in the subiculum (n = 20; 43.2%), CA4 (n = 17; 14.4%), CA1 (n = 11; 9.3%), CA3 (n = 10; 8.5%) and CA2 (n = 9; 7.6%). Referring to the slices classified as HS type 2, three patterns of epileptiform activity were observed: (1) interictal-like events (n = 6; 54.5%); (2) periodic ictal spiking (n = 3; 27.3%); and (3) seizure-like events (n = 2; 18.2%). Induction-susceptibility of epileptiform activity was predominant in the dentate gyrus (n = 5; 45.5%), followed by that in CA4 (n = 2; 18.2%), subiculum (n = 2; 18.2%), CA1 (n = 1; 9.1%) and CA2 (n = 1; 9.1%); however, no activity was observed in the CA3 region. Regarding HS type 3, four patterns of epileptiform activity were observed: (1) interictal-like events (n = 3; 42.9%); (2) periodic ictal spiking (n = 2; 28.6%); (3) spreading depression-like events; and (4) seizure-like events (n = 1; 14.3%). The only two regions that generated activity were the subiculum (n = 4; 57.1%) and the dentate gyrus (n = 3; 42.9%) (Fig. 4). Finally, statistical analysis was performed to assess subfield-specific histopathological data and clinical parameters with electrophysiological data, but no significant differences were observed.

### Epileptiform activity is characterized by different frequencies in each hippocampal subfield

**Figure 5:**
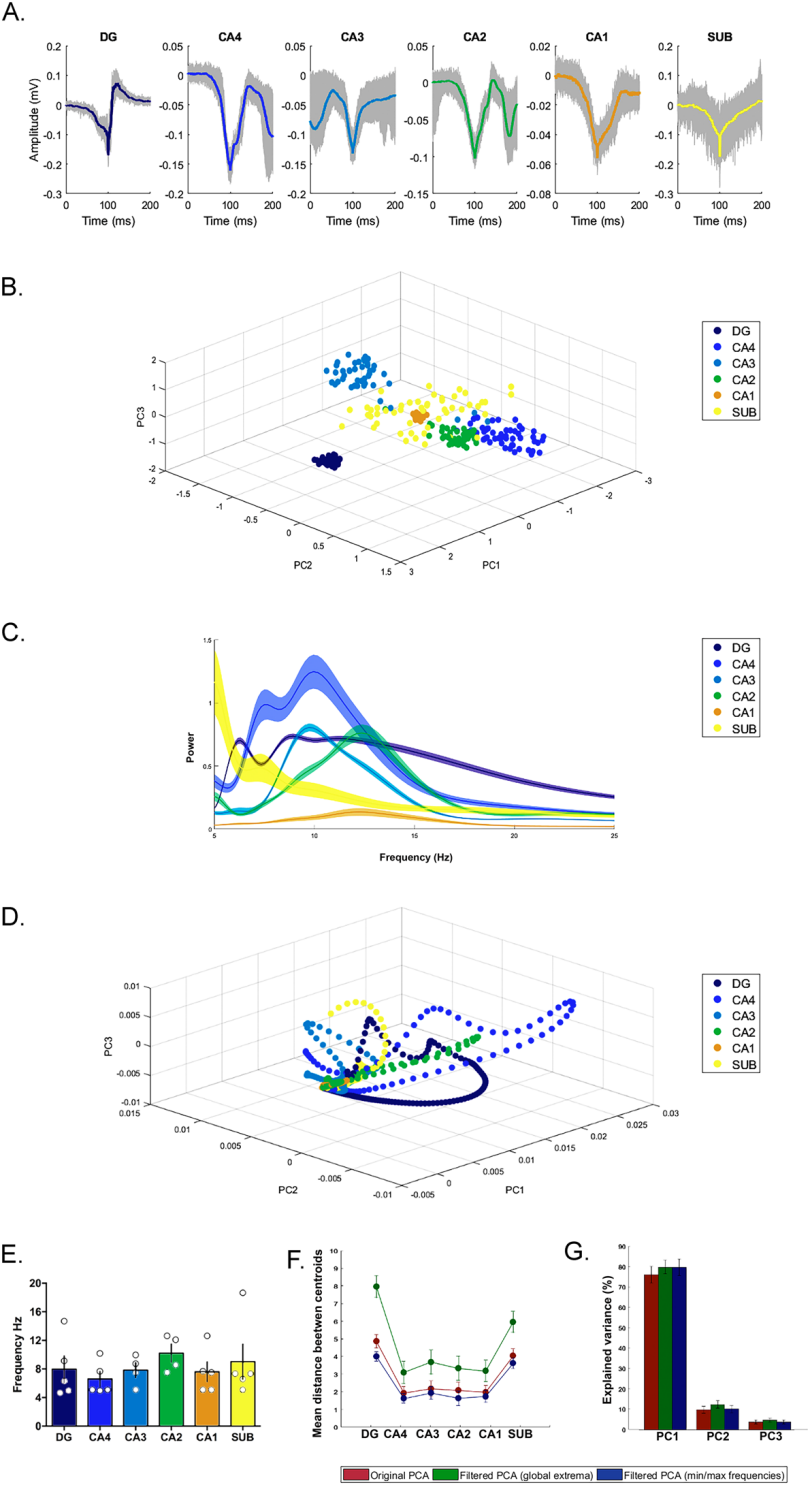
Electrophysiological signal analysis. Electrophysiological analysis of one illustrative patient (case one) is showed in (**A**–**D**). Analysis of the other four patients also selected in this study are found in the supplementary information. (**A**) Averaged waveform of epileptiform patterns in each hippocampal subfield was obtained using the spike sorting-like procedure. The waveform in color represents the average of trial recordings, with 95% confidence intervals (gray background). (**B**) Principal component analysis showing different clusters among hippocampal subfields. (**C**) Power frequency of the different hippocampal subfields; note that each subfield exhibited different power-frequency ranges. The average power frequency is represented by the continuous line in color with their respective confidence interval of α = 0.05. (**D**) Principal component analysis applied on the frequency domain; observe the different trajectories and critical points in each hippocampal subfield. (**E**–**G**) shows analysis of all five tissue samples. (**E**) Maximal frequencies displayed by the epileptiform patterns in different hippocampal subfield. Bar graphs represent the means and standard deviation. (**F**) Cluster centroid distances in each hippocampal subfield between original PCA (red circles) and PCA after filtering recordings using the critical points in each trajectory (green circles) and with minimal and maximal frequencies (blue circles). The distance centroid is represented by the confidence interval mean and its standard deviation. (**G**) Percentage of explained variances of PCA with filtered and unfiltered data. Bar graphs represent the mean and standard deviation.

We performed electrophysiological analysis of interictal-like events and periodic ictal spiking recorded in hippocampal slices of five patients, who were selected because they presented these patterns in most of their hippocampal formation areas (slices n = 21; epileptiform patterns n = 48). An illustrative case of one patient is showed in Fig. 5 (and the remaining 4 cases are showed in the supplementary information).

The average waveforms of epileptiform-like activity in each hippocampal formation area is shown in Fig. 5A. The clusters shown in Fig. 5B, generated by PCA analysis, highlight the differences in waveform yielded by each hippocampal subfield from each tissue sample. Since a waveform pattern is a result of a combination of different frequencies, it is reasonable to assess how cluster formation is affected by different frequency ranges. As expected, the predominant hippocampal frequency range was the theta rhythm, as shown by the power spectrum in Fig. 5C and E. However, each subfield also exhibited high power at certain frequencies. Therefore, to evaluate correlations among these frequencies, PCA was applied on the time-frequency matrices. The trajectories shown in Fig. 5D, obtained by PCA, express the sequential changes of the waveform pattern according to each wavelet scale. At low wavelet scales, no distinction was observed among subfield responses, which formed a single cluster. As the frequency pattern increases (wavelet scale compression), different trajectories start to appear, and critical points (scales that generate shift on the curves) were observed, suggesting optimal frequency ranges. Finally, to evaluate if these critical points express optimal responses, each subfield signal was filtered considering the global critical point associated with each trajectory. A significant increase in the Euclidean distance between the cluster centroid was observed when comparing the results of all five tissue samples before and after the filtering procedure (Fig. 5F). Figure 5G displays the variances explained by PCA for each principal component, wherein an increase in the filtered components for the global critical point was observed, but it was not statistically significant. Taken together, these findings suggest an intrinsic role of frequency ranges in the electrophysiological responses of the different hippocampal subfields of sclerotic tissue.

## Discussion

We investigated population activities of the subiculum, CA1, CA2, CA3, CA4 and the dentate gyrus in sclerotic hippocampi of 30 patients with drug-resistant TLE. To the best of our knowledge, this is the first study to demonstrate experimentally induced self-sustained epileptiform activity in the CA1, CA3 and CA4 subfields which exhibit severe neuronal cell loss in the sclerotic human hippocampus. This study also demonstrated an association between hippocampal subfields and generation of specific patterns of epileptiform activity, which are as follows: (1) CA3 and CA4 with interictal-like activity, (2) subiculum and CA2 with periodic ictal spiking, (3) dentate gyrus with seizure-like events. In addition, we observed that induced epileptiform-like activity exhibits frequency-specific ranges in each subfield of the sclerotic human tissue.

Previous studies using *in vitro* electrophysiology of human brain slices from sclerotic hippocampi have demonstrated epileptiform like-activity in the dentate gyrus, subiculum and CA2^18,19,22–24^. In the dentate gyrus, we observed five patterns of epileptiform activity: interictal-like event, periodic ictal spiking, spreading depression-like events, tonic seizure-like events and seizure-like events, which is consistent with the research literature^23^. Gabriel and colleagues (2004) only assessed the dentate gyrus subfield in their study; however, our investigation stands out by comparing the incidence of ictal activity in the dentate gyrus with the remaining hippocampal regions, thereby allowing us to observe that this region exhibited the highest susceptibility to generate epileptiform-like activity. We also verified the predominance of seizure-like events in the dentate gyrus of HS slices. In addition to HS slices, Gabriel *et al*.^23^ studied non-HS slices demonstrating that seizure-like events and periodic ictal spiking were exclusively generated in human HS slices. These authors suggested that occurrence of these epileptiform-like activities is possibly the result of aberrant circuitry due to network reorganization of the human sclerotic hippocampus; mossy fiber sprouting and the delayed rectifier potassium current (I_k_) were considered to be important contributors to the hyperexcitable population responses^23,28,29^.

The subiculum–an anatomical area between the hippocampus and the entorhinal cortex–is the major output structure of the hippocampus. The subiculum has been suggested as a potential zone for the origin of rhythmic discharges since spontaneous interictal activity has been recorded in this region through *in vitro* recordings, in both sclerotic and non-sclerotic hippocampi excised from patients with pharmacoresistant TLE. These observations suggest that hippocampal sclerosis is not mandatory for the establishment of an epileptic focus in this region (Cohen *et al*.^19^; Wozny *et al*.^24^). However, we were unable to record spontaneous activity in the subiculum, and hence, K-aCSF was used to induce epileptiform-like activity in the subiculum. Several studies using different experimental approaches to induce ictal-like discharges in slices from human subjects with epilepsy reported the generation of seizure-like activity in the subiculum area^22,30,31^. In our study, in addition to seizure-like activity, we also observed periodic ictal spiking, interictal like-events and spreading depression-like events in this brain area, possibly due to the induction protocol used in our experiments. However, it is important to note that a significant association between the subiculum and periodic ictal spiking events was observed. In non-pathological conditions, the subiculum presents a robust local intrinsic connectivity among pyramidal neurons making them susceptible to recurrent excitation, which may be highly exacerbated in the epileptic condition. This hypothesis may be supported by a previous report demonstrating that in human TLE associated with hippocampal sclerosis, the isolated subiculum is able to generate interictal-like activity by itself^19^. Depolarizing GABAergic signaling, through alterations in chloride homeostasis, may also account for hyperexcitability in the subiculum^20,32^.

As demonstrated for the subiculum, our study also encountered a significant association between CA2 and periodic ictal spiking, possibly involving different underlying mechanisms. In CA2, reorganization of the mossy fiber excitatory synapses, associated with depolarizing GABAergic responses due to functional alterations in perisomatic-targeting parvalbuminergic interneurons, seems to be responsible for the generation of this pattern of epileptiform-like activity^18^. In our study, despite subjecting pathological tissue to induction of epileptiform discharges, the CA2 region did not generate epileptiform activity, such as seizure-like events, spreading depression-like events or even tonic seizure-like events, possibly due to the high expression of calcium binding proteins found in this region^17,33^.

The results of our experiments demonstrate, probably for the first time, that epileptiform-like activity may be induced in CA1, CA3 and CA4 in human brain slices *in vitro*. Other studies also addressed this issue by investigating CA1, CA3, dentate gyrus and subiculum using multi-electrode recordings in human hippocampal slices; however, they failed to observe spontaneous activity in the CA1, CA3 and dentate gyrus^19,22^. In our study, the hippocampus proper (CA1-CA4) exhibited the lowest susceptibility to generate epileptiform-like activity. In addition, we observed that both the CA3 and CA4 subfields were associated with the occurrence of interictal-like activity. Similar results were observed in a previous study^34^, wherein *in vivo* experiments in rats subjected to the pilocarpine model of epilepsy demonstrated that occurrence of two types of interictal spikes prevailed in the CA3 region. Using high extracellular potassium in rat brain slices, the CA3 subfield generated interictal-like activity; however, it was resistant to generate seizure-like events^35^. Studies in the CA3 subfield in slices of pilocarpine-epileptic rats have demonstrated rare spontaneous bursts in this region but have also described an increased sensitivity to generate bursts in the presence of high extracellular potassium concentration compared to that in control slices^36^. In CA3, interictal-like events under the excitatory influence of high extracellular potassium seem to be a product of the intrinsic burst properties of pyramidal neurons and their strong recurrent excitatory synaptic connections^37,38^.

Unlike CA3, CA1 pyramidal neurons exhibit comparably little excitatory connectivity with each other under non-pathological conditions, hence the basis of their synchronization relies on CA3^39^. In hippocampal sclerosis, CA1 pyramidal cells are highly vulnerable to neuronal death likely due to poor control mechanisms to contain neuronal excitability. Although it is evident that CA1 may easily generate epileptic activity, the role that this region plays in TLE is still unknown because the pyramidal cells of this region are virtually absent in the sclerotic hippocampus which has been suggested as the ictogenic focus in TLE^39^. *In vitro* studies analyzing the CA1 region using rodent brain slices are greater in number than those for other subfields of the hippocampus, because it is easier to keep the cells alive and relatively easy to obtain electrophysiological recordings in this subfield^40^. In our study, it was difficult to access the field potentials of the CA1 area, and of the other subfields of the hippocampus proper, possibly due to severe neuronal loss and reduction of the laminar organization of neuronal cells in the preparation of slices. However, we recorded four patterns of epileptiform-like activity in the CA1 subfield: interictal-like events, periodic ictal spiking, spreading depression-like events and seizure-like activity, but without statistical association between area and activity. Previous studies have suggested that the wide regional variation in neuronal loss may explain why potassium-buffering ranged from normal to severely altered within the CA1 area of distinct hippocampal slices from epileptic rats (Gabriel *et al*.^41^). In addition, spatial reduction in potassium-buffering has been described in the CA1 subfield of sclerotic slices from patients with epilepsy as well as in pilocarpine-treated epileptic rats (Gabriel *et al*.^42^). Other investigations performed in slices of pilocarpine-treated epileptic rats have indicated the increased excitability in the CA1 area^43^, possibly due to modifications in GABAergic inhibitory interneuron connectivity as well as the presence of aberrant sprouting axons in this region, which would contribute to increased excitability and propagation of epileptiform-like activity via augmented backward excitation^44–46^.

The CA4 is a hippocampal subfield that possesses a diversity of neuron types^47^; however, few studies have been dedicated to investigating their electrophysiological properties. Mossy cells are the most abundant neurons in this subfield; intracellular recordings in rat hippocampal slices have demonstrated these neurons as having predominant excitatory synaptic effects on granule cells^48,49^. Moreover, it was also described that mossy cells receive extensive inputs from granule cells via mossy fiber collaterals. In our study, we observed two types of epileptiform-like activity in the CA4 hippocampal subfield: seizure-like events and interictal-like events. Although we did not address the origin of epileptiform activity from the different types of neurons in the CA4 area, the presence of activity in this region could be facilitated by the severe loss of parvalbumin-interneurons^50^ that would lead to disinhibition of principal cells as well as the recurrent inputs of mossy fiber sprouting onto mossy cells. Additionally, it has been described that neuronal networks with only few neurons are capable of generating and sustaining epileptiform activity, possibly by spreading the activity of a single neuron towards several neurons via multisynaptic excitatory pathways that lead to synchronal burst activity in these areas that are affected by severe neuronal loss^51,52^.

Concerning our findings regarding frequency-specific bands of epileptiform activity generated by hippocampal sectors, we suggest that each subfield of hippocampal formation works as an independent nucleus of information processing, where the hippocampus, through a subfield network, processes information in parallel and rhythmically but also distinctly. Our hypothesis is that this is a physiological characteristic of the hippocampus, wherein the epileptic condition of the tissue would exacerbate or modulate this characteristic. Therefore, considering that an epileptiform signal is generated by hypersynchronization of specific groups of neurons, which amplify extracellular potentials, the epilepsy tissue assessed in our study would maximize the responses of each subfield, hence, explaining their intrinsic electrophysiological characteristics. The idea that brain regions process information at different frequencies has been widely debated. Studies in rat hippocampal slices described gamma oscillations generated independently in the CA1 area, by induced excitability through pharmacological manipulation, and these oscillations persisted even when connections from CA3 and the entorhinal cortex were severed^53^. This gamma rhythm was also observed in the subiculum area^54^. Other studies have described that the hippocampus presents different types of neuronal discharging in different frequency bands^55^. At the macroscale level, studies have described that frequency-specific interactions observed in different brain areas are necessary for efficient coordination in linguistic processing^56^. In our study, we also observed specific frequencies generated by each hippocampal subfield, which is in accordance with previous investigations. We observed that theta oscillations had frequency-power predominance, for most subfields; however, each subfield presented differences in their power frequency regime of oscillation, suggesting a structure-functional distinctiveness. Therefore, taking together the previous investigations and our findings, we propose that each hippocampal subfield generates unique frequency oscillations which play a role in hippocampus communication, not only in physiological but also in epileptiform pathological signaling.

On the other hand, our histopathological data is in agreement with previous studies which demonstrate that severe neuronal loss is predominant in the CA1 and CA4 hippocampal subfields, and that the HS type 1 is the most frequent pattern of neuronal loss^3,57^. Morphological data in our study was used to compare neuronal density and dentate gyrus width with electrophysiological data; however, non-statistical differences were observed, possibly due to the small sample sizes of HS types 2 and 3.

Finally, the present study has certain limitations. The difficulties we encountered in addressing the underlying mechanisms responsible for the epileptiform activity generation in each hippocampal formation area were probably associated with the implicit limitations of working with human epileptic tissue (lack of control tissue, damage of neural connections due to excision procedure and slicing, absence of spontaneous epileptiform activity). Nevertheless, we were able to demonstrate that epileptiform-like activity may be induced in different regions of the hippocampal formation, including regions that are severely affected by neuronal loss in TLE associated with hippocampal sclerosis. These findings obtained from human tissue may contribute to future studies investigating the specific role of the different hippocampal subfields in the generation of epileptiform activity in patients with pharmacoresistant TLE and to obtain insights into the dynamic networks involved in seizure generation.

## Methods

### Subjects

Thirty human sclerotic hippocampal specimens surgically resected from patients with pharmacoresistant TLE, during the period from 2014 to 2017, were included in the study. The study group consisted of 19 (63%) women, 11 (37%) men, with a mean age of 32 ± 12 years (details of clinical features in Table 2). Hippocampal specimens (n = 12) obtained from autopsies of individuals without history of neurological diseases or known brain abnormalities were used as age-matched controls for histopathological analysis.

All patients underwent presurgical evaluation including prolonged video electroencephalogram monitoring, high-resolution magnetic resonance imaging, neuropsychological and quality of life assessment. Written informed consent was obtained from all patients before epilepsy surgery at São Paulo Hospital, in Brazil. The study was approved by the Ethics Committee of Universidade Federal de São Paulo (CAAE 47551015.1.0000.5505) and procedures were conducted in conformity with the Declaration of Helsinki for human experimentation.

### Hippocampal tissue preparation

Immediately following hippocampal resection, the tissue was incubated in carbogenated transport solution (4 °C) containing the following: 3 mM KCl, 1.25 mM NaH_2_PO_4_, 10 mM glucose, 2 mM MgSO_4_, 2 mM MgCl_2_, 1.6 mM CaCl_2_, 21 mM NaHCO_3_, 200 mM sucrose, and 0.1 mM (±) a-tocopherol pre-dissolved in ethanol (pH 7.4, osmolality 303 mOsm/kg); samples arrived at the laboratory ten minutes later. Next, using a vibratome (Leica VT 1200S), the tissue was sectioned coronally into slices with thickness of 500 µm. Approximately 4–5 slices were obtained for electrophysiological recording and the remaining tissue was subjected to immunohistochemistry analysis. Slices were transferred to an interface chamber with a perfusion rate of 1.7–2.0 ml/min with prewarmed carbogenated artificial cerebrospinal fluid (aCSF) at 34.5 ± 0.5 °C containing the following: 129 mM NaCl, 3 mM KCl, 1.25 mM NaH_2_PO_4_, 10 mM glucose, 2 mM MgSO_4_, 1.6 mM CaCl_2_, 21 mM NaHCO_3_, and 0.03 mM (±) a-tocopherol, adjusted to pH 7.4 (osmolality of 303 mOsm/kg). To allow maximal recovery of the brain tissue after surgery, recordings were taken 4–5 hours after slice preparation.

### Electrophysiological Recordings

Extracellular recordings were performed in the granule cell layer of the dentate gyrus and in the pyramidal cell layer of the CA1, CA2, CA3, CA4 and the subiculum, using six independent extracellular glass electrodes simultaneously (filled with 154 mM NaCl, tip diameter 2–3 µm, resistance 2 to 4 MΩ), placed 150 μm below the surface of the slice. To confirm slice viability, paired stimuli (0.1 ms, 1–30 V, 50 ms interval) were applied at the beginning and at the end of each experiment through a bipolar electrode (20 µm diameter, platinum wires, tip separation 60–100 µm) positioned at the CA4/CA3 border. Slices displaying maximal population spike amplitudes of up to1.5 mV in the dentate gyrus at the beginning, or amplitude loss greater than 20% of the maximal amplitude at the end of the experiment, were excluded from analysis.

Epileptiform activity was induced in the dentate gyrus by a single 15-min period of hilar stimulation and continuous perfusion with aCSF containing 10–12 mM [K^+^] (high K-aCSF). The hilar stimulation consisted in paired stimuli given at every 20-sec (during 15-min), evoking field responses with 80% of the maximum population spike amplitude, previously determined by input-output relationship of stimulus intensity versus population spikes. When epileptiform activity was observed independently of electrical stimulation, the hilar stimulation was turned off. In the subiculum and hippocampal subfields, high levels of K-aCSF alone were sufficient to provoke epileptiform activity.

Signals were amplified using a custom-made amplifier equipped with capacitance and offset potential compensation, filtered at 3 kHz, digitized online and stored for offline analysis using Spike 2 v6.09 (CED-1401, United Kingdom), with a sample rate of 10 kHz. Rates (events per minute), durations (from onset up to two-thirds recovery of the shift field potential) and amplitudes (low-frequency component and peak-to-peak amplitude) of events were measured during 10 minutes after the stabilization of the event rate and amplitude of the epileptiform activity in each hippocampal formation area.

### Categorization of epileptiform-like activity

In this study, the epileptiform events were categorized as previously described^11,23^, which was as follows: (1) interictal-like events: negative field potential transient of short duration (0.21–2.6 sec), with event rates <40/min; (2) periodic ictal spiking: very short negative discharges with event rates >40/min; (3) seizure-like events: slow negative field potential shifts (>5 sec) with initial burst and subsequent high-frequency fluctuations of field potentials followed by low frequency clonic-like discharges; (4) tonic seizure-like event: characterized by recurrent long-lasting (15–162 sec) negative field potential shifts, without superimposed clonic-like discharges; (5) spreading depression-like event: large change of the slow negative field potential with amplitudes >1 mV.

### Neuropathological evaluation

Resected hippocampal tissue was cut at a thickness of 5 mm, fixed in 10% formalin for 24 hours and subsequently embedded in liquid paraffin^58^. A single block from each patient was sliced at a 5-µm thickness and sections were immunostained against neuron-specific nuclear protein (NeuN, monoclonal, 1:1000, Chemicon). Hippocampal subfields (CA1, CA2, CA3 and CA4) and subiculum were analyzed at four and ten randomly selected microscopic sectors (each 250 × 250 µm) respectively, with an objective magnification of 20 × and neuronal cells were manually counted. The cell count of each hippocampal area was expressed as the mean number of neurons x 10^−4^/µm^2^ corresponding to a representative sampling for the entire hippocampus. In the dentate gyrus, the internal and external limbs were investigated at 10 arbitrarily chosen microscopic fields with 20 × augmentation to estimate the width of the granule cell layer. Neuronal cell counting and estimation of the dentate gyrus width were performed using the software ImageJ. Cell densities and granule layer width were converted into z-score values obtained from age-matched autopsy controls as previously reported^59–61^.

The pattern of neuronal loss was classified according to the *International League Against Epilepsy* (ILAE) criteria: (a) type 1, predominant neuronal loss in CA1 and CA4 hippocampal subfields; (b) type 2, predominantly affecting CA1; (c) type 3, cell loss predominant in CA4^3^. The histopathological patterns of dentate gyrus were classified as: (a) preserved (b) cell loss (c) dispersion or (d) bilamination^62^.

### Data analysis

#### Statistics

The Shapiro-Wilk test was applied to verify normality origin of the data and to determine a correspondent parametric or nonparametric test. For pairwise comparisons of non-parametric tests, the Wilcoxon test was used. Contingency analysis using the chi-square test was performed to evaluate the association between categorical data. For group comparisons, analysis of variance (ANOVA-one way) or its non-parametric version, the Kruskal-Wallis test, was used to compare and evaluate the different parameters of epileptiform activity among different regions. All patient data were grouped according to the histopathological diagnosis, averaged and compared, to assess the possible impact of epilepsy (age at epilepsy-onset, duration, initial precipitating injury, seizure frequency, AED, more details in Table 2). The significance level for all analyses was established using α = 0.05.

#### Electrophysiological signal analysis

**Figure 6:**
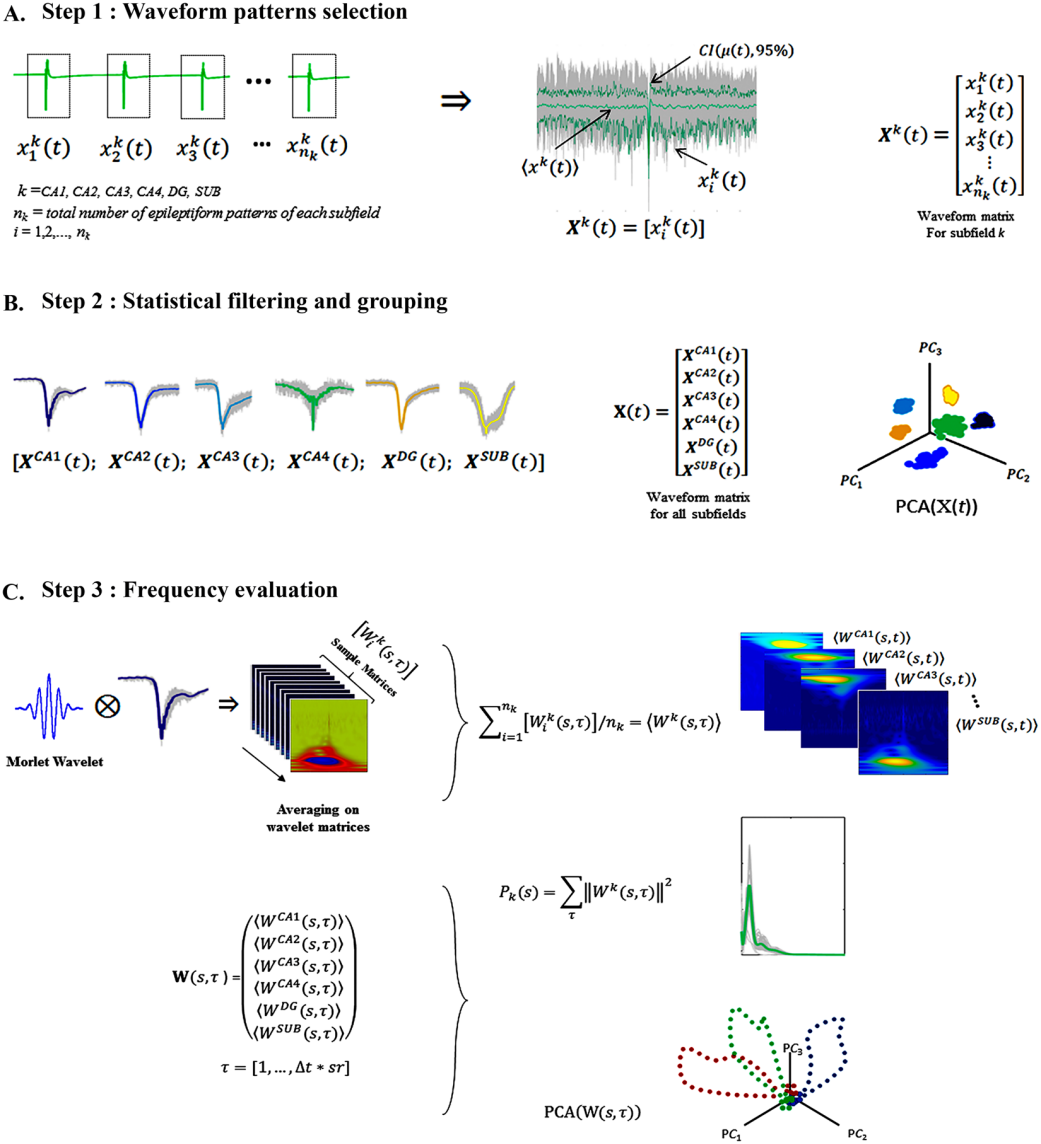
Methods for electrophysiological signal analysis. (**A**) By spike sorting-like procedure, epileptiform patterns were selected and averaged by confidence intervals (α = 0.05). (**B**) After obtaining the waveform matrices, PCA was applied to the global matrix ${\rm{X}}(\tau )$. (**C**) For frequency domain analysis, each epileptiform pattern was processed by the wavelet technique, an average of sample matrices and wavelet-matrices for each subfield was obtained. The power spectrum was calculated from the global time-scale matrix, and PCA was applied.

First, electrophysiological signals were processed using Butterworth filters with an order of 20 and 60 Hz notch. Periodic ictal spiking and interictal-like events were identified along the electrophysiological recordings for each hippocampal subfield ($k\,=\,$CA1, CA2, CA3, CA4, dentate gyrus and subiculum). By using a spike sorting-like procedure^63^, ${n}_{k}$ sample patterns ${x}_{i}^{k}(\tau )\,$for each *k* hippocampal subfield, were cut and time-locked considering the minimum peak of the activities as reference, yielding pieces of patterns with $\tau \,=\,$20  ms. Next, the average ${x}^{k}(\tau )$ of the trials ${x}_{i}^{k}(\tau )\,$was calculated and the outliers were removed using the minimum width envelope method^64^ and defining a confidence interval $CI(\alpha =0.05)\,$of the cumulative trials $[{x}_{i}^{k}(\tau )]$ (Fig. 6A).

#### Waveform analysis

Principal component analysis (PCA) was applied onto the global matrix ${\rm{X}}(\tau )$, composed by all subfield waveform matrices ${X}^{k}(\tau )={[{x}_{i}^{k}(\tau )]}_{i=1,\ldots ,{n}_{k}}$, to identify cluster differences and similarities among all patterns generated by each hippocampal subfield (Fig. 6B).

#### Power frequency characterization

To detect differences in the frequency domain among hippocampal subfields, each trial of the time-locked patterns ${x}_{i}^{k}(\tau )\,$was processed using the wavelet technique, since the signals are not stationary, thereby emphasizing complex patterns at different scales^65^. In this process, each trial was down-sampled to 5000 Hz and the time-frequency spectrum was calculated using a continuous wavelet technique with mother wavelet Morlet, with 9 octaves and 32 voices to cover the 5–2500 Hz band. Therefore, for each trial *i* of each subfield *k*, ${x}_{i}^{k}(\tau ),$a set of time-scale matrices ${{W}_{i}^{k}(s,\tau )|}_{i=1,\ldots ,{n}_{k}}$ was constructed. Finally, the wavelet power spectrum ${P}_{k}(s)$ was calculated by taking the absolute-value squared of the average scale-matrices ${W}^{k}(s,\,\tau )$ coefficients, and summing and normalizing the time domain (Fig. 6C).

#### PCA of epileptiform patterns

To evaluate correlations in the frequency domain, the PCA technique was applied on the global wavelet time-scale matrix ${\rm{W}}(s,\tau )$ composed by the concatenation of all hippocampal subfield average matrices ${W}^{k}(s,\tau )$, where Δ*t* = 200 ms and the sampling rate *sr* = 5 kHz (Fig. 6C).

## Electronic supplementary material

Supplementary information
