# Supplementary material for: Different patterns of epileptiform-like activity are generated in the sclerotic hippocampus from patients with drug-resistant temporal lobe epilepsy
Source: Sci Rep. 2018 May 8;8:7116. doi: 10.1038/s41598-018-25378-9 (PMC5940759; doi:10.1038/s41598-018-25378-9)
Supplement: Supplementary file 1 — Supplementary information [file 41598_2018_25378_MOESM1_ESM.pdf]

Different patterns of epileptiform-like activity are generated in the sclerotic hippocampus from patients with drug-resistant temporal lobe epilepsy

Selvin Z. Reyes-Garcia[1,2]*, Carla A. Scorza[1], Noemi S. Araújo[1], Nancy N. Ortiz-Villatoro[1], Anaclara Prada Jardim[1], Ricardo Centeno[1], Elza Márcia Targas Yacubian[1], Jean Faber[1], Esper A. Cavalheiro[1]

[1] Departamento de Neurologia e Neurocirurgia, Escola Paulista de Medicina, Universidade Federal de São Paulo, São Paulo, Brazil.

[2] Departamento de Ciencias Morfológicas, Facultad de Ciencias Médicas, Universidad Nacional Autónoma de Honduras, Tegucigalpa, Honduras.

* Corresponding author: selvin.reyes@unah.edu.hn

Supplementary information:

Electrophysiological analysis of interictal-like events and periodic ictal spiking recorded in hippocampal slices of four patients, who were selected because they presented these patterns in most of their hippocampal formation areas.

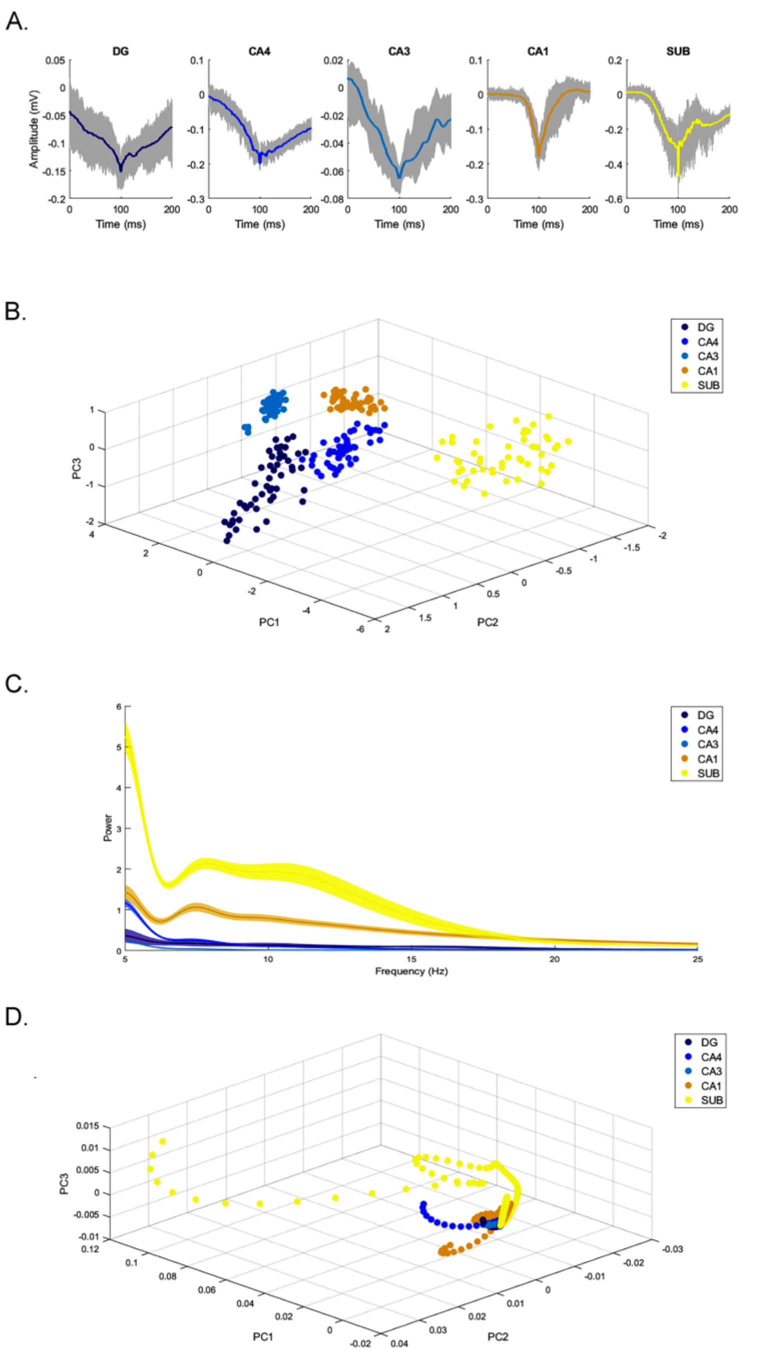

Supplementary Figure S1. Case two.

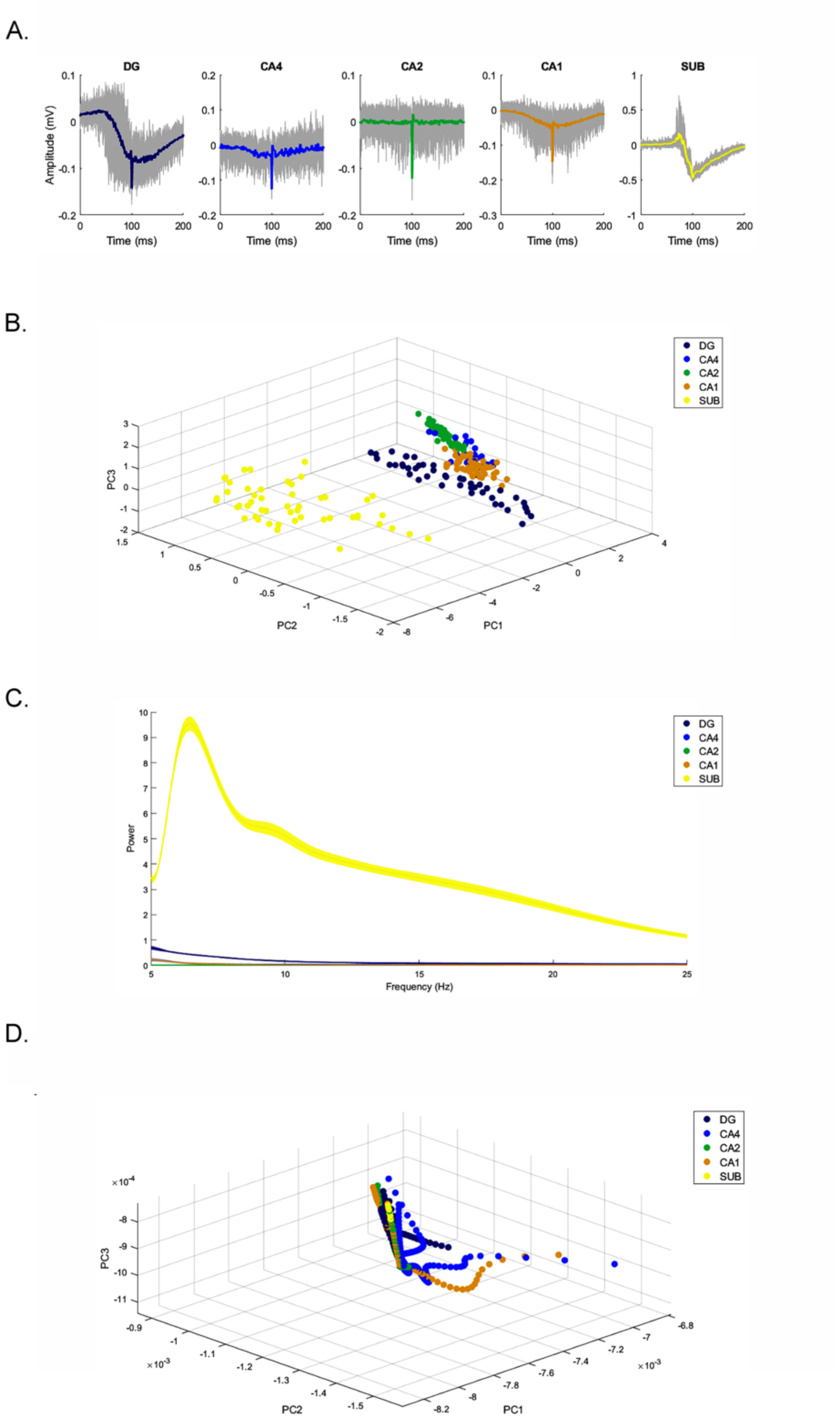

Supplementary Figure S2. Case three.

A.

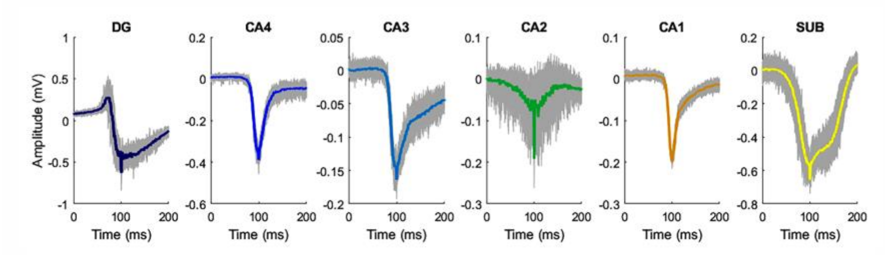

B.

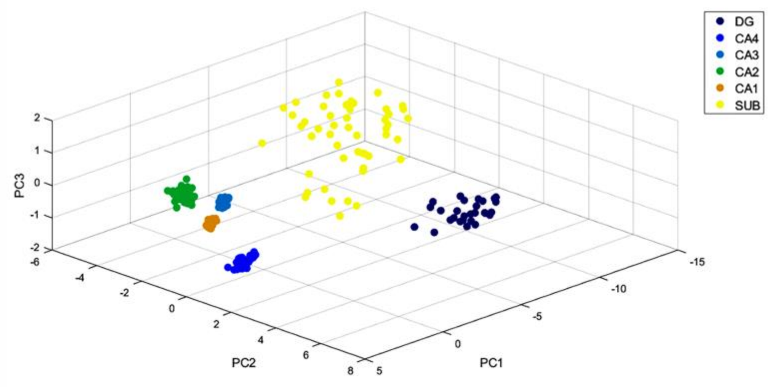

C.

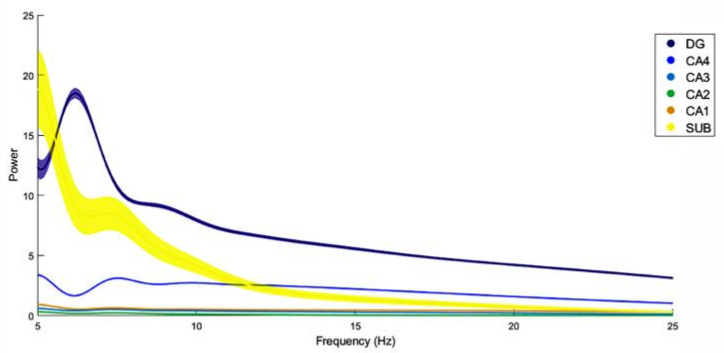

D.

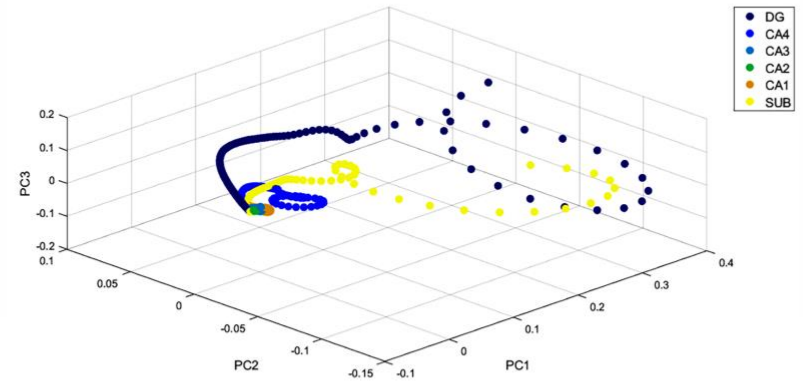

Supplementary Figure S3. Case four.

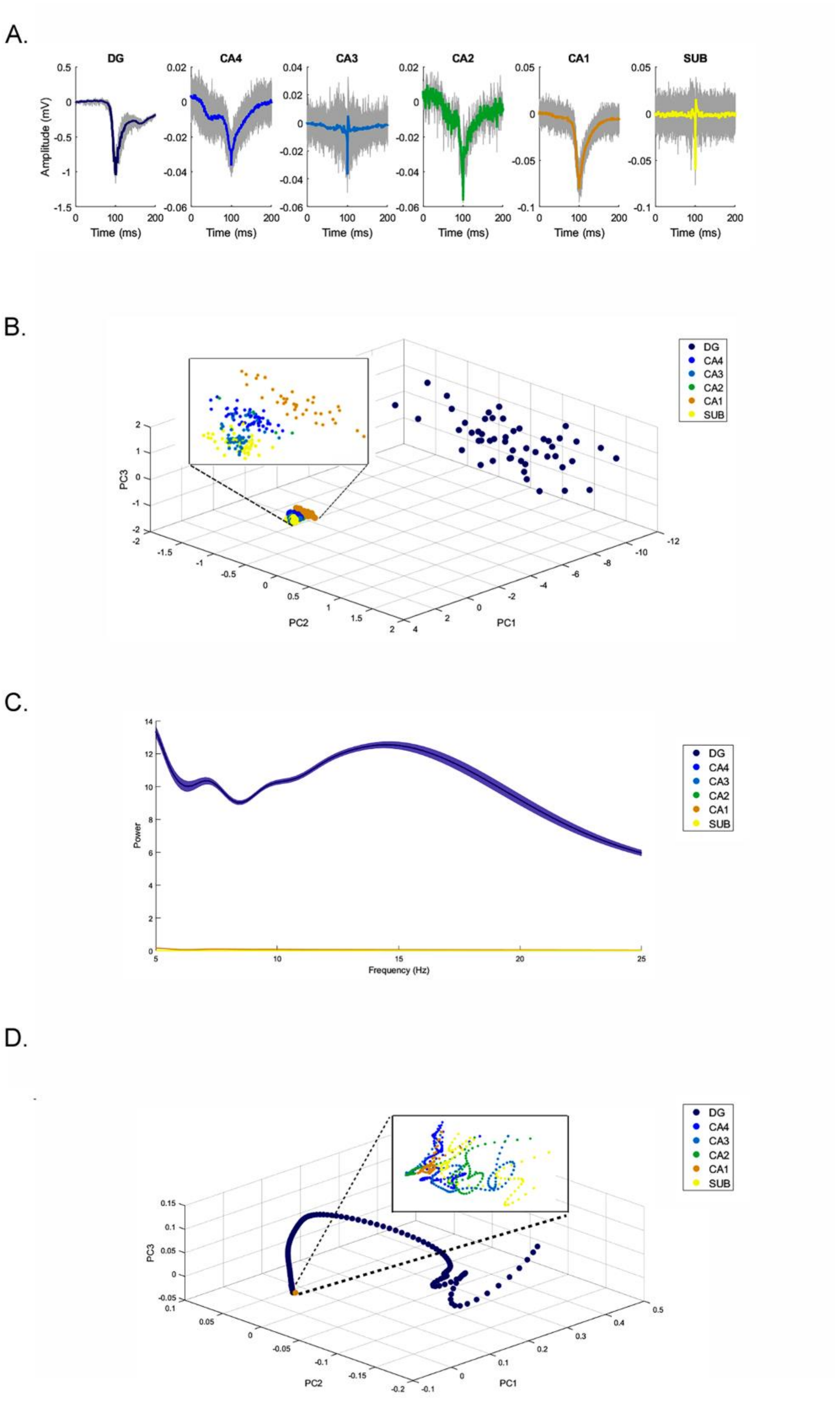

Supplementary Figure S4. Case five.

Electrophysiological signal analysis of cases 2, 3, 4 and 5. (A) Averaged waveform of epileptiform patterns in each hippocampal subfield was obtained using the spike sorting-like procedure. The waveform in color represents the average of trial recordings, with 95% confidence intervals (gray background). (B) Principal component analysis showing different clusters among hippocampal subfields. (C) Power frequency of the different hippocampal subfields; note that each subfield exhibited different power-frequency ranges. The average power frequency is represented by the continuous line in color with their respective confidence interval of α=0.05. (D) Principal component analysis applied on the frequency domain; observe the different trajectories and critical points in each hippocampal subfield. In cases 2 and 3, no activity was observed in CA2 and CA3, respectively.
